# 24‐Hour postnatal total serum protein concentration affects the health and growth performance of female Holstein dairy calves

**DOI:** 10.1002/vms3.1203

**Published:** 2023-07-17

**Authors:** Mitra Aghakhani, Amir Davar Foroozandeh Shahraki, Seyed Nouroldin Tabatabaei, Majid Toghyani, Erfaneh Moosavi‐Zadeh, Hassan Rafiee

**Affiliations:** ^1^ Department of Animal Science, Isfahan (Khorasgan) Branch Islamic Azad University Isfahan Iran; ^2^ Animal Science Research Department, Isfahan Agriculture and Natural resources Research and Education Center Agriculture Research, Education and Extension Organization (AREEO) Isfahan Iran

**Keywords:** body structures, dairy calves, growth, health, total serum protein

## Abstract

**Background:**

Total serum protein (TSP) within the first few days of life in the neonatal calf has predictive value for subsequent growth and production in calves before and after weaning.

**Introduction:**

The objective of this study was to evaluate the effect of TSP concentration 24‐h after birth (24‐h) on the performance and health of Holstein dairy calves.

**Methods:**

A total of 152 female calves were enrolled in this study. Blood samples were collected at 24‐h, and TSP concentration was measured with a refractometer. Calves based on TSP concentration at 24‐h were allocated into three groups: 1 – TSP ≤6.5 g/dL, 2 – TSP between 6.6 and 6.9 g/dL and 3 – TSP ≥7 g/dL. The weighing was done at birth and at days 30 and 60. Starter feed intake was recorded from day 3 to weaning, and body structures were measured at birth and weaning day.

**Results:**

Calves with TSP >6.5 g/dL had greater body weight at days 30 and 60 than calves with TSP ≤6.5 g/dL. Average daily gain during 1–30 and 1–60 days of life increased as TSP increased. Furthermore, starter feed intake during the first 60 days of life was greater in calves with TSP ≥7 g/dL than calves with TSP <7 g/dL. The TSP concentration influenced structural growth, and >6.5 g/dL calves had greater heart girth, hip width and body length than ≤6.5 g/dL calves. Moreover, odds ratio for pneumonia decreased as TSP increased, whereas diarrhoea was unaffected.

**Conclusion:**

The TSP at 24‐h is an important contributing factor for the variation in growth performance and health of preweaning calves, and strategies to improve calf immunity and increase TSP lead to better animal health during preweaning period.

**Implications:**

These results indicated that TSP ≥6.5 g/dL possibly was associated with greater performance, and this concentration could be considered a baseline for future analyses.

## Graphical abstract

Total serum protein (TSP) within the first few days of life has predictive value for subsequent growth andproduction of dairy calf. Calves with TSP >6.5 g/dL had greater starter intake and growth during the first 60 d of life. These results indicated that TSP >6.5 g/dL could be considered a baseline for future. analyses.

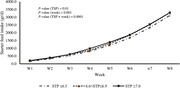

## 1 INTRODUCTION

Bovine colostrum is defined as first secretion after parturition and contains higher protein, immunoglobulins, non‐protein nitrogen, fat, vitamins and minerals compared to normal milk (Kelly, 2003). Due to the absence of transfer of antibodies by placental blood to the calf during gestation, the transfer of passive immunity through the absorption of antibodies from colostrum is essential for neonatal calves. Therefore, colostrum management and providing high‐quality colostrum in proper time and volume are well recognized as vital in preventing disease during a period that the immune system will gradually activate in neonatal calves (Godden et al., 2019). Colostrum quality is well defined by immunoglobulin G (IgG) concentration less than 2.0 g/dL as poor quality, 2.0–5.0 g/dL as moderate quality and finally the greater than 5.0 g/dL as excellent quality (Shearer et al., 1992). The current recommendation is the intake of colostrum containing the IgG concentration >5.0 g/dL during the first 6–8 h of life. It is well demonstrated that colostral IgG concentration will peak approximately 24–36 h after feeding colostrum (Hassig et al., 2007). Therefore, if the serum IgG concentration exceeded 1.0 g/dL during that time, calves to be relatively well protected against pathogens (McGuirk & Collins, 2004). In a recent study, four serum IgG categories were proposed including excellent, good, fair and poor with serum IgG levels of ≥2.5, 1.8–2.49, 1.0–1.79 and <1.0 g/dL, respectively (Lombard et al., 2020). These researchers showed a reduction in morbidity and mortality rates with increasing serum IgG levels.

Total serum protein (TSP) or IgG concentration within the first few days of life is the primary indexes for the failure of passive transfer (FPT) (Godden et al., 2019). In the neonatal calf, IgG constitutes a large proportion of the protein in serum. This allows the measurement of TSP to provide an estimation of serum IgG concentration (Morrill & Tyler, 2012). The TSP measurement is more practical in farm conditions, as well as highly correlated with blood IgG concentration in calves (Thornhill et al., 2015). Many articles used refractometers for measuring the TSP (Cuttance et al., 2019; Hue, Williams, et al., 2021; Hue, Skirving, et al., 2021; Vogels et al., 2013). Hue, Williams et al. (2021) reported that serum protein refractometer measurements predicted serum IgG level with high accuracy, providing an on‐farm test to determine that calves have received sufficient passive immunity and colostrum components.

Moreover, passive transfer of immunity has predictive value for subsequent growth and production in calves before and after weaning (Denise et al., 1989; Elsohaby et al., 2019; Robinson et al., 1998). The FPT was associated with underdevelopment of the digestive tract and lower feed intake, resulting in reduced growth rates during rearing, and decreased milk production during the first lactation, compared with calves that had adequate passive immunity (DeNise et al., 1989; Donovan et al., 1998; Robison et al., 1988). For example, a significant association was indicated between serum IgG concentration after birth with body weight (BW) and average daily gain (ADG) during the first 21 (Elsohaby et al., 2019) and 180 (Robison et al., 1988) days of life in dairy calves. Robison et al. (1988) reported that mortality of heifer calves through 6 months of age with FPT was 6.78% compared with 3.33% mortality for calves with adequate passive immunity. In addition, calves that survived with FPT failed to grow rapidly through 180 days of age. Furthermore, calf serum Ig concentration 24–48 h after birth was a significant source of variation in mature milk and fat production, and heifers with FPT had lower milk production during their first lactation (Denise et al., 1989).

In the previous studies, the suggested cut‐off values for the FPT using a refractometer were <5.2 g/dL TSP in healthy calves (Tyler et al., 1996; Windeyer et al., 2014) and <5.5 g/dL TSP in clinically ill calves (McGuirk & Collins, 2004; Tyler et al., 1996). Furthermore, Donovan et al. (1998), in a large study with 3300 female calves, indicated the lowest risk of calves’ mortality and morbidity until 6 months of life was associated with TSP >6.5 g/dL, and suggested this level will be considered the baseline for future analyses. Moreover, Manriquez et al. (2018) categorized concentrations of TSP as low (≤6.5 g/dL); medium (6.6–7.4 g/dL); or high (≥7.5 g/dL). Furthermore, Lombard et al. (2020) reported in a systematic review that calves with TSP ≥6.2 g/dL have an excellent transfer of passive immunity. Based on these studies (Donovan et al., 1998; Lombard et al., 2020; Manriquez et al., 2018), it seems that calves with TSP >6.5 g/dL have an excellent transfer of passive immunity and lowest risk of mortality and morbidity until 6 months of life and suggesting that TSP >6.5 g/dL will be considered the baseline for future analyses. We categorized concentrations of TSP as low (≤6.5 g/dL); medium (6.6–6.9 g/dL); or high (≥7.0 g/dL) for evaluating the TSP concentration on calves’ performance in the current experiment.

However, to our knowledge, there is limited information about the association between TSP concentration at 24‐h and BW, ADG, starter feed intake, body structure growth and morbidity in the preweaning period for dairy calves. Therefore, this study aimed to assess the effects of calves’ TSP concentration during the first 24 h after colostrum feeding on health, growth performance and starter feed intake of female Holstein dairy calves.

## 2 MATERIALS AND METHODS

### 2.1 Experimental design, cow management and treatments

The experiment was carried out in a large commercial dairy herd, consisting of 3500 lactating cows in the Isfahan district during autumn 2018 with mean temperature and relative humidity 7°C and 51%, respectively.

For this experiment, a total of 152 female Holstein dairy calves (39.0 ± 4.7 kg of initial BW), without birth defects, having been delivered without assistance, and showing a regular respiratory pattern, were eligible for enrolment. It was suggested in the literature that TSP ≥6.5 g/dL would be considered the baseline for future analyses, and the calves based on TSP concentration at 24 h of life (24‐h) were allocated into three experimental groups: 1 – TSP concentration ≤6.5 g/dL, 2 – TSP concentration between 6.6 and 6.9 g/dL and 3 – TSP concentration ≥7 g/dL.

The calves were separated within 10 min after birth, not allowed to suckle on their dams, weighed and moved to individual pens (1.22 × 2.44 m) with sawdust bedding. Calves were weighed on an electronic scale and fed maternal colostrum based on an average of 10% of their birth weight, approximately 30 min after separation from their dam. If all colostrum was not consumed, the remainder was fed 2 h later after birth. According to farm protocol, the colostrum was harvested within 30 min postpartum using a portable milking machine. Colostrum of individual cows was milked into clean buckets and fed to their calves in bottles according to their BW. Colostrum feeding was followed by feeding 3 L of transitional milk per day until day 3. The transitional milk from all dams was pooled and all calves fed transitional milk with similar quality. In the current experiment, transition milk is the milk that cows produce after colostrum during second and third milkings. After that, calves received whole milk (mean composition: 3.6% ± 0.13% fat, 3.2% ± 0.01% CP, 4.8% ± 0.15% lactose and 12.8% ± 0.52% total solids) in galvanized buckets twice daily at 0800 and 1500 h until weaning. All calves were fed 5 L/day of milk from day 4 to 15 and offered 6 L/day of milk from day 16 to 21 of age and then 7 L/day from day 22 to 50 and 4 L/day from day 51–56 and 2 L/day from day 57–60 (total milk offered = 331 L).

At day 4, calves were fed starter feed containing 224 g/kg CP, 39 g/kg ether extract and 11.79 MJ/kg ME on a DM basis prepared as a mashed form. Starter feed was formulated according to CNCPS and calves had free access to starter feed by offering an amount that resulted in the residue of 10% of offered feed after 24 h. Through the experiment, calves had free access to the starter feed and fresh water from the nipple.

### 2.2 Sampling and measurements

BW was measured at birth, days 30 and 60 of life about 4 h after morning milk feeding. Starter refusals were recorded and renewed every day at 10:00 AM. The starter feed intake of each calf was calculated from the difference between the daily starter feed that was offered and refused. Means of ADG were determined.

Body measurements of each calf include body length (distance between the points of shoulder and rump), withers height (distance from the base of the front feet to the withers), body barrel (circumference of the belly before feeding), heart girth (circumference of the chest), hip height (distance from the base of the rear feet to hook bones) and hip‐width (distance between the points of hook bones) were measured with calipers at the birth and weaning (day 60) according to the method described by Mirzakhani et al. (2021).

Calves were monitored individually twice daily (before milk feeding) for any signs of ill thrifty, such as weakness, decreased appetite, dehydration, fever, abnormal faecal consistency, increased respiratory rate and other lethargy signs. Faeces were scored daily according to the procedure of Habibi et al. (2020) and categorized as firm and well‐formed (score 1), soft and pudding‐like (score 2), runny and pancake batter (score 3) and liquid and splatters (score 4). A diarrheic calf was considered if the faecal score was ≥3. If having a faecal score ≥3 and coughing or nasal discharge, the calves were examined by the veterinarian to confirm diarrhoea or pneumonia (Habibi et al., 2020). The treatment procedures for diarrhoea and pneumonia were applied at the FKA Milk and Meat Co. (Isfahan). Treatments were discontinued in calves that did not respond to the treatment. No animal died during the study.

### 2.3 Blood sampling and measurements

Blood samples were taken approximately at 24‐h after birth from the jugular vein into untreated evacuated tubes. The blood samples were centrifuged at 1500 × *g* for 15 min at 4°C, and the serum was analysed using an optical protein refractometer (Instrument‐Model PA203; US patent D510880) for total protein. TSP was measured twice using a refractometer, and the mean of these measurements was recorded. Distilled water was used to calibrate the refractometer before each use.

Colostrum samples were collected in 50 mL in plain evacuated serum tubes (BD Vacutainer, Becton, Dickinson and Co., Belliver Industrial Estate) per cow for IgG analyses by ELISA sandwich method (Bovine IgG ELISA Bioassay Tech. Lab E0010Bo Kit, assay sensitivity of 1.03 μg/mL and Intra and inter‐CV were <8.00% and <10.00%).

### 2.4 Experimental design and analysis

At first, a linear regression was used to assess the linear relationship between TSP concentration at 24‐h with performance traits (BW at day 60 and ADG).

To evaluate the effect of TSP on calf performance factors (growth traits and starter feed intake) linear mixed model (PROC MIXED) was used. For traits such as starter feed intake with repetitions during the experiment, they were analysed as repeated measurement. Also, to estimate the odds ratio of disorders due to differences in TSP at 24‐h used a logistic regression model with PROC GLIMMIX assumed a binary distribution with the logit link function. All statistical analysis was conducted using the SAS/STAT 9.4 (SAS Institute Inc.). Additionally, the differences among the treatments were evaluated using a multiple comparison test following the Tukey–Kramer method. Statistical significance of any main effect was declared at *p* ≤ 0.05. The statistical model used for the analyses was as follows:

*Y*_ijklm_ = *μ* + TP*_i_* + Period*_j_* + *β*_1_ × Bweight*_k_* + *β*_2_ × Bstra*_l_* + Calf*_m_* + TP*_i_* × Period*_j_* + *e*_ijklm_

where *Y*_ijklm_ is the dependent variable (weight as continuous; disorders as binomial trait; starter feed intake as continuous; and structure growth traits as continuous); TP*_i_* is the TSP at 24‐h (three levels; 1: ≤6.5 g/dL; 2: between 6.5 and 6.9 g/dL; and 3: ≥7.0 g/dL) as an independent variable, period*_j_* is the measurement time, *β*_1_ and *β*_2_ are regression coefficient for birth weight and birth structure traits (e.g. heart girth and withers height) and Bweight*_k_* and Bstra*_l_* are covariates of birth weight and birth structure, respectively, TP*_i_* × period*_j_* is the interaction of TSP and measurement time, Calf*_m_* is the random effect of calf and *e*_ijkl_ is the random residual error.

## 3 RESULTS

**FIGURE 1:**
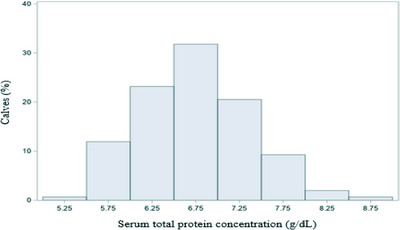
Distribution of total serum protein concentration (g/dL) at 24 h of life of 152 female Holstein calves.

Results of descriptive analysis for TSP concentration at 24‐h, colostrum's IgG concentration, birth weight and BW at days 30 and 60 of Holstein dairy calves are presented in Table 1. TSP concentration at 24‐h ranged from 5.3 to 8.9 g/dL, colostrum's IgG concentration ranged from 45.2 to 116.5 mg/mL, birth weight ranged from 25 to 50 kg, BW at day 30 ranged from 33 to 69 kg and BW at day 60 ranged from 41 to 91 kg. Furthermore, the distribution of TSP concentration at 24‐h of 152 calves used in the current study is presented in Figure 1.

**TABLE 1:** Descriptive statistics for 152 Holstein calves enrolled in the current study.

Items	Mean (± SD)	Minimum	Maximum
Total serum protein at 24 h (g/dL)	6.7 ± 0.6	5.3	8.9
Colostrum's IgG (mg/mL)	84.1 ± 15.0	45.2	116.5
Birth weight (kg)	39.0 ± 4.7	25.0	50.0
Body weight at day 30 (kg)	48.3 ± 7.4	33.0	69.0
Body weight at day 60 (kg)	70.9 ± 10.1	41.0	91.0

The results of linear regression analysis between TSP and calf growth performance showed that for each unit increase of TSP, the final weight of the calf increased by 6.53 kg (CI: 3.86–7.91; *p* < 0.001), and ADG increased by 23 g/day (CI: 16.9–26.8; *p* = 0.04, Table 2).

**TABLE 2:** Results of the final linear relationship for serum total protein and calves’ growth performance.

Variables	Estimate (± SE)	*p* Value
Body weight at 60 day (kg)		
Intercept	27.00 ± 7.80	0.007
Total serum protein	6.53 ± 1.15	<0.001
Average daily gain (g/day)		
Intercept	603.61 ± 162.25	0.003
Total serum protein	23.04 ± 14.61	0.04

**FIGURE 2:**
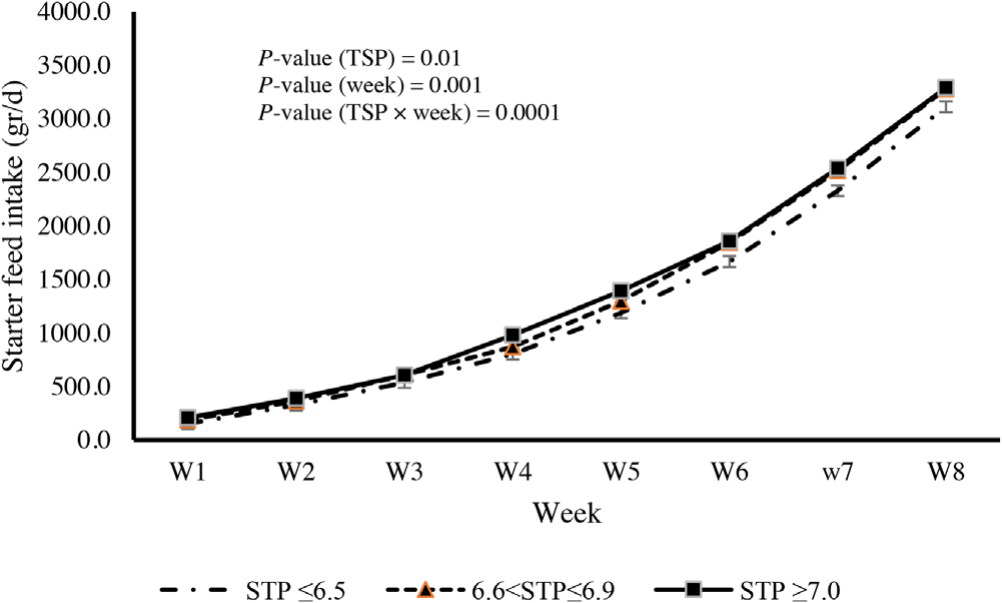
Starter feed intake during the first 8 weeks of life with three levels of total serum protein (TSP) at 24 h of life.

As shown in Table 3, calves with TSP >6.5 g/dL at 24‐h had greater BW at 30 (*p* = 0.001) and 60 (*p* = 0.001) day of life than calves with TSP ≤6.5 g/dL at 24‐h. Furthermore, ADG during 1–30 and 1–60 days of life increased as TSP increased, whereas ADG was greater during 30–60 days of life in calves with TSP ≥7 g/dL than calves with TSP <7 g/dL. Moreover, starter feed intake during the first 60 day of life was greater in calves with TSP ≥7 g/dL than calves with TSP <7 g/dL, and there was no significant difference between calves with TSP ≤6.5 g/dL and between 6.6 and 6.9 g/dL (Figure 2).

**TABLE 3:** Least squares means (± SE) for the effects of total serum protein at 24 h of life on starter feed intake, average daily gain, feed efficiency and body weight of dairy calves.

	total serum protein at 24 h of life (g/dL)			
Items	≤6.5	6.6–6.9	≥7.0	*p* Value
Birth body weight (kg)	38.7 ± 0.45	39.4 ± 0.53	39.9 ± 0.53	0.11
Body weight at day 30 (kg)	45.63 ± 0.49^b^	49.70 ± 0.50^a^	50.20 ± 0.56^a^	0.001
Body weight at day 60 (kg)	69.31 ± 0.90^b^	71.38 ± 1.04^a^	72.83 ± 1.02^a^	0.001
Average daily gain (g/day)				
1–30 days	220.6 ± 16.7^c^	355.9 ± 19.2^b^	373.1 ± 18.2^a^	0.001
31–60 days	754.7 ± 25.4^b^	723.5 ± 28.9^b^	789.5 ± 29.9^a^	0.04
1–60 days	505.0 ± 15.0^c^	539.5 ± 17.0^b^	563.7 ± 17.0^a^	0.04
Starter feed intake (g/day)				
1–30 days	461.4 ± 14.2^b^	456.1 ± 16.6^b^	536.5 ± 15.0^a^	0.003
31–60 days	2067.9 ± 33.0^b^	2073.5 ± 39.0^b^	2282.0 ± 36.0^a^	0.001
1–60 days	1264.6 ± 18.4^b^	1264.5 ± 21.9^b^	1409.0 ± 20.9^a^	0.001

^a‐c^Means within a row with different superscripts are significantly different (p < 0.05).

The results of structural growth are listed in Table 4. The TSP at 24‐h influenced structural growth, and >6.5 g/dL calves had greater heart girth, hip width and body length than ≤6.5 g/dL calves. Body barrel was greater in ≥7.0 g/dL calves than ≤6.5 g/dL calves, but 6.6–6.9 g/dL was not different from ≥7.0 to ≤6.5 calves. Moreover, increasing TSP increased withers height and hip height.

**TABLE 4:** Least squares means (± SE) for the effects of total serum protein at 24 h on body structures measurements (cm) of dairy calves at 60 days of life.

	Total serum protein at 24 h (g/dL)			
Items	≤6.5	6.6–6.9	≥7.0	*p* Value
Heart girth	96.32 ± 0.61^b^	98.81 ± 0.41^a^	99.82 ± 0.68^a^	0.001
Body barrel	106.07 ± 0.7^b^	108.10 ± 0.92^ab^	109.61 ± 0.89^a^	0.001
Withers height	84.21 ± 0.40^c^	85.94 ± 0.46^b^	87.74 ± 0.44^a^	0.001
Hip height	88.88 ± 0.42^c^	91.09 ± 0.49^b^	92.57 ± 0.47^a^	0.003
Hip width	14.42 ± 0.16^b^	16.71 ± 0.19^a^	16.90 ± 0.18^a^	0.01
Body length	51.61 ± 0.40^b^	53.08 ± 0.46^a^	53.73 ± 0.44^a^	0.001

^a‐c^ Means within a row with different superscripts are significantly different (p < 0.05).

Moreover, odds ratio for pneumonia between birth and weaning decreased as TSP increased (Table 5). The TSP concentration at 24‐h had no significant effect on the incidence risk of diarrhoea in dairy calves.

**TABLE 5:** Estimated odds ratios (95% CI) for the effects of total serum protein at 24 h on diarrhoea and pneumonia of dairy calves during first 60 days of life.

	Total serum protein at 24 h (g/dL)			
Disorders	≤6.5	6.6–6.9	≥7.0	*p* Value
Diarrhoea				
Incidence risk (%)	55.17	55.80	50.0	0.42
OR (± CI)	Reference	0.98 (0.67–1.19)	0.78 (0.50–1.21)	
Pneumonia				
Incidence risk (%)	10.34^a^	3.42^b^	1.60^c^	0.001
OR (± CI)	Reference	0.13 (0.04–0.47)	0.05 (0.01–0.20)	

^a‐c^ Means within a row with different superscripts are significantly different (p < 0.05).

## 4 DISCUSSION

The objective of this study was to investigate the association between TSP at 24‐h and growth performance, starter feed intake, body structural growth and health in preweaning dairy calves. This study demonstrated that TSP concentrations at 24‐h <6.5 g/dL were associated with increased calf morbidity and reduced ADG, BW, structural growth and starter feed intake during the preweaning period. Furthermore, these results suggested that TSP >6.5 g/dL could be considered a new baseline for future analyses. In the present study, the TSP concentrations were measured by a refractometer. Many articles used refractometers for measuring the TSP (Cuttance et al., 2019; Hue, Williams, et al., 2021; Vogels et al., 2013; Hue, Skirving, et al., 2021). Hue, Williams et al. (2021) reported that serum protein refractometer measurements predicted serum IgG level with high accuracy, providing an on‐farm test to determine that calves have received sufficient passive immunity and colostrum components.

The TSP concentration at 24‐h positively influenced days 30 and 60 BW, day 60 body structure growth and ADG from birth to day 60. These results suggest that passive transfer status significantly affects growth performance during the first month of life (Mastellone et al., 2011). In agreement with our finding, Mastellone et al. (2011) detected significant linear associations between serum Ig concentration at 24‐h and BW at day 30 and ADG from birth to day 30 in buffalo calves. Moreover, a significant association was indicated between serum IgG concentration after birth with BW and ADG during the first 21 (Elsohaby et al., 2019) and 180 (Robison et al., 1988) days of life in dairy calves. Moreover, Cuttance et al. (2018) showed that the weaning weights increased by 0.4 kg for every 1.0 g/dL increase in TSP concentration. Stefańska et al. (2021) reported that BW was associated with initial TSP concentration between 24 and 48 h after birth, and calves with greater TSP had greater BW and ADG until day 450 of life. Furman‐Fratczak et al. (2011) stated that TSP levels did not affect ADG for the first 6 months, but claves with greater TSP levels had greater ADG between 12 and 15 months of life and achieved BW allowing first insemination 17 day sooner. The increase in ADG and BW of calves possibly is attributed to greater starter feed intake in the current study. Thus, improving colostrum management practices in dairy farms and greater TSP in calves could increase starter feed intake and ADG. Starter feed intake is an important factor for ruminal development and minimizing weaning BW slump and stress, and for adapting to future solid diets, which could also directly influence future performance (Silva et al., 2019). Moreover, colostrum is a source of hormones and growth factors, including insulin, IGF‐I, IGF‐II, epidermal growth factor, growth hormone, adiponectin and leptin (Bach, 2012). These factors promote the development of the gastrointestinal tract and the production of digestive enzymes (Guilloteau et al., 1997). Therefore, the adequacy of colostrum and IgG intake has both short‐ and long‐term effects on dairy calves (Shivley et al., 2018) and probably influence their future growth rate, fertility and milk production. On the other hand, it was reported that ADG reduced by 0.067 kg/day in calves experienced calfhood respiratory disease (Buczinski et al., 2021) and calves with lower TSP concentration experienced more pneumonia in the current study.

To our knowledge, this is the first experiment that examines the effect of TSP at 24‐h on preweaning starter feed intake and body structural growth. Calves fed greater starter feed received more energy and protein, resulting in greater ADG and body structure. Generally, starter feed is an important part of a preweaned calf's diet, and the aim is that the calf is consuming enough starter to sustain a good ADG and body growth during the preweaning period. However, there is a common concern that an increase in BW can be attributed to greater gut fill and increased weights of gastrointestinal tissues not the frame size of body (Khan et al., 2011). Calves are expected to increase in BW concomitantly with structural growth, and skeletal growth is equally important function to consider because those body dimensions are not often influenced by body condition, degree of fatness or gut fill (Heinrichs et al., 1992). Because 50% of withers height increase occurs in the first 6 months of life (Kertz et al., 1998), we anticipated that the calves with greater TSP g/dL would gain faster than calves with TSP <6.5 g/dL in both BW and frame size. Although mature body size is determined by genetic potential, but diet and health status can result in animals achieving that genetic potential earlier or being retarded in growth and never achieving their maximal mature size (Owens et al., 1993). Thus, the increased structural growth of calves with TSP greater than 6.5 g/dL might be the result of additional energy and nutrients available for skeletal deposition due to the increase in DMI and these calves may have the potential to achieve structural maturity at a younger age.

Calves fed adequate colostrum immunoglobulin may develop and grow normally, as opposed to calves with failure of transfer of passive immunity, which have decreased nutrient utilization and reduced feed intake (Colditz, 2004; Massimini et al., 2007). A meta‐analysis by Soberon et al. (2012) found that every  kg increase in preweaning ADG and could increase the first lactation milk yield by 1550 kg. It could be hypothesized that manipulation of heifer TSP level may offer a viable method of increasing first lactation milk yield through greater starter feed intake, ADG and body structural growth during the preweaning period. In agreement with our results, Stefańska et al. (2021) showed that the initial TSP concentration of dairy heifers was associated with greater withers height and lower age at first oestrus, artificial insemination service, pregnancy and calving and improvement actual and energy‐corrected milk during first lactation.

Calfhood diseases have a major impact on the economic viability of dairy cow operations, due to the direct costs of calf losses and treatment and the long‐term effects on performance. The lower incidence of diseases due to greater TSP concentration was expected because of the great impact of colostrum‐derived immune cells on calves’ health. Many of non‐immunoglobulin factors in colostrum (i.e. cytokines, GH and IGF‐1) might have interacted with Ig concentration or acted directly to influence the growth response or to advance the immune and metabolic systems of the dairy calves (Hagiwara et al., 2000; Reber et al., 2005). The same significant association was reported in previous studies (Nocek et al., 1984; Robison et al., 1988; Virtala et al., 1996). Cuttance et al. (2018a) reported that calves with TSP <5.2 g/dL had 1.68 times higher odds of experiencing a farmer‐recorded animal health event between birth and weaning. In agreement with our results, Donovan et al. (1998) and Stefańska et al. (2021) noted TSP was not associated with incidence, age of onset or severity of diarrhoea. However, pneumonia was strongly associated with TSP during the first 6 months (Donovan et al., 1998). Virtala et al. (1996) reported that each additional week of pneumonia reduced total BW gain and total height gain during the first 3 months of life by 0.8 kg and 0.2 cm, respectively. Moreover, higher mean haptoglobin levels and higher intensity and frequency of respiratory and gastrointestinal tract morbidity were observed in the calves with lower Ig levels at 30–60 h of life (Furman‐Fratczak et al., 2011). An increase in haptoglobin concentration is commonly related to the intensity of an inflammatory reaction (Heegaard et al., 2000). Probably calves with greater TSP have a greater capacity to inactivate pathogenic invasion and mount an earlier response than calves with lower TSP; the latter must mount an acquired humoral immune response for defence (Elsohaby et al., 2019). Prevention of diseases (such as pneumonia) in the early life of dairy calves by increasing the TSP level may improve ADG and structural growth of calves. Together, our results and previous studies indicate that strategies to improve calf immunity and increase TSP lead to better animal health during the preweaning period, irrespective of the dairy management system.

The initial feeding (colostrum and transitional milk quality and quantity) is an important factor in calf performance and health. In the present study, the calves received their dam's colostrum at the rate of 10% of their BW. In the following, the calves received similar transition milk, whole milk and the starter feed, and it could be noted that the most important factor affecting the difference in TSP level was the quality of colostrum consumed. The colostrum's IgG concentration ranged from 4.52 to 11.65 g/dL. In agreement, Immler et al. (2022) and Turini et al. (2020) reported TSP increased in association with an increase in the Brix percentage of the colostrum administered to the calves and calves being fed colostrum with higher Brix values had greater TSP concentration.

## 5 CONCLUSIONS

Our results suggest that TSP at 24‐h had positive effects on BW, ADG, starter feed intake, body structural growth and lower pneumonia during the preweaning period. Moreover, TSP at 24‐h is a significant source of variation in growth performance and morbidity during the preweaning period. Calves with TSP >6.5 g/dL had greater starter feed intake and lower pneumonia incidence, leading to greater ADG and body structural growth during the preweaning period. Our results indicate that strategies to improve calf immunity and increase TSP lead to better animal health during the preweaning period. Moreover, our findings suggested that TSP >6.5 g/dL could be considered a new baseline for future analyses.

## AUTHOR CONTRIBUTIONS

*Conceptualization; formal analysis; methodology*: Mitra Aghakhani. *Conceptualization; funding acquisition; investigation; supervision*: Amir Davar Foroozandeh Shahraki. *Investigation; supervision*: Seyed Nouroldin Tabatabaei and Majid Toghyani. *Methodology*: Erfaneh Moosavi‐Zadeh. *Writing original draft; writing review and editing*: Hassan Rafiee.

## CONFLICT OF INTEREST STATEMENT

The authors declare that there are not any conflicts of interest.

## ETHICS STATEMENT

Guidelines for the care and use of animals were approved by the Iranian Council of Animal Care (1995). The authors confirm that the ethical policies of the journal, as noted on the journal's author guidelines page, have been adhered to and the appropriate ethical review committee approval has been received. The authors confirm that they have followed EU standards for the protection of animals used for scientific purposes.

### PEER REVIEW

The peer review history for this article is available at https://publons.com/publon/10.1002/vms3.1203.

## Data Availability

The data that support the findings of this study are available from the corresponding author upon reasonable request.
